# The Hypoxic Ischemic Encephalopathy Model of Perinatal Ischemia

**DOI:** 10.3791/955

**Published:** 2008-11-19

**Authors:** Hidetoshi Taniguchi, Katrin Andreasson

**Affiliations:** Department of Neurology and Neurological Sciences, Stanford University School of Medicine

## Abstract

Hypoxic-Ischemic Encephalopathy (HIE) is the consequence of systemic asphyxia occurring at birth. Twenty five percent of neonates with HIE develop severe and permanent neuropsychological sequelae, including mental retardation, cerebral palsy, and epilepsy. The outcomes of HIE are devastating and permanent, making it critical to identify and develop therapeutic strategies to reduce brain injury in newborns with HIE.  To that end, the neonatal rat model for hypoxic-ischemic brain injury has been developed to model this human condition. The HIE model was first validated by Vannucci et al ^1^ and has since been extensively used to identify mechanisms of brain injury resulting from perinatal hypoxia-ischemia ^2^ and to test potential therapeutic interventions ^3,4^.  The HIE model is a two step process and involves the ligation of the left common carotid artery followed by exposure to a hypoxic environment.  Cerebral blood flow (CBF) in the hemisphere ipsilateral to the ligated carotid artery does not decrease because of the collateral blood flow via the circle of Willis; however with lower oxygen tension, the CBF in the ipsilateral hemisphere decreases significantly and results in unilateral ischemic injury. The use of 2,3,5-triphenyltetrazolium chloride (TTC) to stain and identify ischemic brain tissue was originally developed for adult models of rodent cerebral ischemia ^5^, and is used to evaluate the extent of cerebral infarctin at early time points up to 72 hours after the ischemic event ^6^. In this video, we demonstrate the hypoxic-ischemic injury model in postnatal rat brain and the evaluation of the infarct size using TTC staining.

**Figure:**
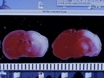


## Protocol

This protocol was approved by the Institutional Animal Care and Use Committee at Stanford University and abides by the National Institutes of Health guidelines for the use of experimental animals.

### Neonatal Rat HIE model

Post natal day (PND) 7 Sprague-Dawley rat pups (Charles River Laboratories, Inc., Wilmington, MA) are fully anesthetized with isoflurane (Aerrane, Baxter,  Deerfield, IL; 3-4% for induction and 1-2% for maintenance).
A small incision is made in the neck (Extra Thin Iris Scissors #14088-10, Fine Science Tools Inc, Foster City, CA) to expose the left common carotid artery (CCA).
The CCA is ligated with a 5-0 silk suture (5-0 silk, Ethicon, Somer, NJ).
The incision is closed with cyanoacrylate adhesive (Elmers Products Inc, Columbus, OH). The incision is covered with tape and anesthesia is stopped. The pups are returned to their dam and allowed to recuperate for 1-2 hours.
Pups are then placed in a hypoxic chamber that contains 8% oxygen balanced with 92% nitrogen for 100 minutes at 37°C. 8% oxygen/92% nitrogen gas(Airgas, Sacramento, CA) flow via a tube into the mouse cage outfitted with a plastic cover. The cover is made to fit to the cage and has two holes of 2 cm in diameter, one of which to receive the tube connected to the 8% oxygen gas tank and another allows the gas to flowout. The chamber is placed in a water bath and is warmed up prior to use and a thermometer inside of it must register 37°C before the start of hypoxia.
At the end of 100 minutes of hypoxia, the pups are returned again to their dam for recovery.
24 hours after the end of hypoxia, pups are euthanized by deep anesthesia with isoflurane. Brain tissue is perfused with cold normal saline, followed by a solution of cold 1% TTC in phosphate buffered saline, pH 7.4 (Sigma Chemical Co, St Louis, MO). TTC must be kept protected from light.
Brains are removed and cooled for 1-2 minutes on ice, and four coronal sections 3 mm apart (levels 1-4) are cut, beginning rostrally at the level of the opticchiasm and infundibulum, corresponding to the bregma 1.8 ~ 2.0 mm level of the adult mouse brain.
Brain slices are immersed in TTC solution and incubated at 37°C for 8 minutes, followed by fixation in 4% paraformaldehyde/PBS overnight. Because TTC is light sensitive, keep slices away from light during this time.
The TTC stained sections are scanned and digitized. Using Image J (NIH, Bethesda, MD), the following are measured for each level: area of infarction (unstained area), the area of the ipsilateral hemisphere, and the area of the contralateral hemisphere. The percent infarcted area per slice is calculated as: (area of infarct/area of ipsilateral hemisphere) or A_1_, A_2_, A_3_, and A_4_. The percent volume of infarct is calculated as the sum (A_1_ +A_2_ + A_3_+ A_4_) * 3mm. Areas of cortical or striatal injury can also be separately quantified in a similar manner.

### Representative Results


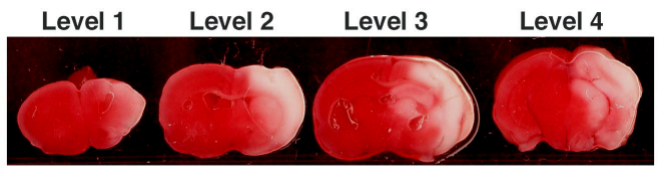
 **Figure 1. Representative HIE coronal levels 1-4 stained with TTC.**

TTC stained coronal sections demonstrate area of infarct (in white).

## Discussion

The rodent postnatal HIE model is an established model that recapitulates cerebral hypoxia occurring in the peri-natal period in newborns.  Extensive histological and immunocytochemical characterization studies have demonstrated that the 7 day old rodent has the analogous brain maturity to model a third trimester human fetus ^7^. The rodent HIE model has been very informative for understanding mechanisms of brain injury from peri-natal hypoxia ^2^, where interventions such as hypothermia have been validated ^3^. This model was initially perfected in rats and more recently adapted to mice ^8^.

There are specific technical details that warrant mention:

Before ligation of the CCA, be sure to remove connective tissue and nervus vagus which may entangle CCA. With successful ligation of the CCA, the vessel color above the ligation site should turn white.  Alternatively, it is also recommended to double ligate and cut in the middle of two ligation site. Cover the incision over the ligated CCA with label tape before placing the pup back with its dam.
A maximum duration of not more than 10 minutes of anesthesia for each pup is optimal for CCA ligation and successful outcome.
The recuperation period after CCA ligation should be 90-120 minutes. If shorter, pups are less likely to survive, and if longer there will be more variability of infarction, tending towards smaller infarcts.
Temperature is critical. Always place a thermometer in the chamber and maintain the temperature at 37°C. The length of hypoxia may vary from 90 to 120 minutes depending on the situation and severity of injury you want to achieve.
TTC staining is applicable for analysis of infarct volume up to 72 hours after HIE.  Longer survival time points should be assessed by sectioning and cresyl violet staining to determine the infarct size.
